# Strain-dependent differences in the capacity of peste des petits ruminants virus to infect antigen-presenting cells

**DOI:** 10.1128/jvi.00294-26

**Published:** 2026-05-27

**Authors:** Aadel Bouziane, Vincent Lasserre, Roger-Junior Eloiflin, Manon Chambon, Camille Piro-Mégy, Samia Guendouz, Philippe Holzmuller, Philippe Totté, Arnaud Bataille

**Affiliations:** 1 CIRAD, UMR ASTRE, Montpellier, France; 2 ASTRE, University of Montpellier, CIRAD, INRAE, Montpellier, France; University of Kentucky College of Medicine, Lexington, Kentucky, USA

**Keywords:** morbillivirus, monocyte-derived macrophage, monocyte-derived dendritic cell, virulence, host susceptibility

## Abstract

The ability of morbilliviruses to infect antigen-presenting cells (APCs) plays a major role in infection outcome, because these cells transport peste des petits ruminants virus (PPRV) from the respiratory tract to the lymph nodes before spreading to lymphoid and epithelial tissues. This study used two cell models—monocyte-derived macrophages (MoMs) and monocyte-derived dendritic cells (MoDCs)—from three host species (goat, sheep, and cattle) to evaluate the differential ability of APCs to be infected by two strains of PPRV with contrasting virulence in goats. Infection was analyzed using flow cytometry, infectious titers (TCID_50_/mL), RT-qPCR, and immunofluorescence. Goat and sheep MoDCs proved highly permissive to both strains, with viral titers at 48 hpi in the range of 10^4^–10^5^ TCID_50_/mL and a high percentage of infected cells. Cattle MoDCs were weakly infected and had no detectable viral titer, as expected for this dead-end host. In comparison, MoMs showed reduced permissiveness to PPRV in goats, but not in sheep. Post-infection supernatants in goat MoMs infected with the low-virulent strain showed low or undetectable TCID_50_/mL, despite persistence of detectable viral genome up to 96 hpi. Immunofluorescence revealed double-stranded RNA but no nucleoprotein of the low-virulent strain in goat MoMs, suggesting a partial engagement of the viral cycle, resulting in a restricted or semi-permissive infection. Overall, these results suggest that this combined *in vitro* model of MoDC and MoM infections would be useful in anticipating host susceptibility and PPRV strain virulence, providing complementary tools for PPR control efforts.

**IMPORTANCE:**

Peste des petits ruminants (PPR) disease affects goats, sheep, and multiple other hosts across the globe, with major impacts on economies, food security, and biodiversity. The sensitivity of host species to PPRV infection varies considerably, with clinical manifestations and disease progression being shaped by the viral strain, host species, and breed infected. *In vitro* models focusing on immune cells, which are the primary target of PPRV, could provide a relevant tool for studying the mechanisms of viral adaptation to the host and cellular antiviral responses, while contributing to surveillance and control strategies. The results obtained here suggest that a dual-cell model focusing on macrophages and dendritic cells could be useful for pre-screening studies aimed at assessing the susceptibility of a given host species to a PPRV strain and estimating its relative virulence. Testing this *in vitro* approach in additional host species and a wider range of strains is required to confirm its relevance.

## INTRODUCTION

Peste des petits ruminants virus (PPRV) is a member of the Paramyxoviridae family, belonging to the *Morbillivirus* genus, which includes the measles virus (MeV), rinderpest virus (RPV), and canine distemper virus (CDV). PPRV has a linear, non-segmented, single-stranded RNA genome with a negative polarity (1). First identified in the Ivory Coast in 1942, PPRV has since spread extensively, becoming endemic in large regions of Africa, the Middle East, and Asia (1, 2), and has recently emerged in several European countries (3, 4). The virus primarily affects domestic small ruminants, such as goats and sheep, but it can also infect suids and a variety of wild species within the order Artiodactyla, including antelopes and gazelles (5).

PPRV is transmitted primarily through direct contact with infected animals, via respiratory secretions, feces, and contaminated feed or water sources. PPR can cause severe clinical manifestations in sheep and goats**,** with high morbidity and mortality, leading to significant economic losses for farmers. Given its high transmissibility and significant economic impact, PPR is classified as a notifiable disease by the World Organisation for Animal Health (WOAH, formerly OIE) (6), and has been the focus of a control program jointly led by WOAH and the Food and Agriculture Organization of the United Nations (FAO) since 2015, aiming for its eradication by 2030 (7). However, the sensitivity of the host species to PPRV infection varies considerably, with clinical manifestations and disease progression being shaped by the viral strain, host species, and breed infected (8, 9). A better understanding of PPRV virulence and host susceptibility is considered a research priority for disease control (10).

The main targets of PPRV and other morbilliviruses are immune cells via the SLAMF1 (Signaling Lymphocytic Activation Molecule Family 1) receptor, also known as CD150 (11, 12), and epithelial cells via the Nectin-4 receptor (13). At the early stage of infection, morbilliviruses infect antigen-presenting cells (APCs), such as macrophages and dendritic cells, which transport the viral particles to lymphoid organs, in particular lymph nodes and tonsils, where lymphocytes reside (1). Infection of epithelial cells occurs once viremia is established, with the Nectin-4 receptor playing an essential role in viral dissemination and excretion (11, 13).

For PPRV, direct infection of APCs *in vivo* has not yet been demonstrated. However, draining lymphoid tissues such as the tonsils and lymph nodes are the first major sites of replication (14). Recent studies have shown that monocyte-derived dendritic cells are permissive to PPRV *in vitro* (15). Permissiveness is defined as the ability of the virus to replicate and produce infectious virions. We know that for other morbilliviruses, permissiveness varies depending on the cell type and the virus infecting these cells (16, 17). Due to the importance of APCs in morbillivirus infection, it is possible that the observed variability in PPRV virulence and host susceptibility can be related to differences in the viral replication capacity in APCs. Among APCs, dendritic cells and macrophages are essential for detecting viral infections and initiating innate immunity (18, 19).

So, the study of infection of monocyte-derived macrophages (MoMs) and monocyte-derived dendritic cells (MoDCs) could be a relevant and reproducible model for comparing the permissiveness of host species to PPRV. Here, we will focus on the infection of MoDCs and MoMs from goats, sheep, and cattle (the latter being identified as dead-end hosts for PPRV [20]) with two strains of PPRV. The two strains used in this study, Morocco 2008 (MA08) and Ivory Coast 1989 (IC89), demonstrated different levels of virulence in certain goat breeds (21). Strain MA08 belongs to lineage IV of PPRV and was isolated from an Alpine goat that was severely affected during the 2008 outbreak in Morocco (22). The IC89 strain belongs to lineage I. It was isolated from a goat in the Ivory Coast in 1989 and is considered to be a mildly virulent strain, inducing a mild disease in various goat breeds (21, 23, 24), although it is used as a virulent strain for the study of immune response in sheep (25).

Based on the representative known virulence to goats of the two strains of PPRV, low for IC89 and high for MA08, the aim of this study was to assess the comparative *in vitro* replication of these PPRV strains in both MoMs and MoDCs of goats, sheep, and cattle. The research question was to evaluate whether any difference could be observed between these APCs in relation to PPRV strain virulence.

## RESULTS

### PPRV infections of MoMs and MoDCs

To determine the infectivity of the two PPRV strains, IC89 and MA08, for macrophages and dendritic cells, circulating monocytes from the blood of the different animal species studied were differentiated into dendritic cells (MoDCs) and macrophages (MoMs). These two cell types were infected with each of the viral strains studied, IC89 and MA08, for 48 hours at a multiplicity of infection (MOI) of 0.1. Infection was assessed by flow cytometry after intracellular labeling with an antibody targeting the virus nucleoprotein (anti-NPPRV) coupled to a fluorochrome.

To identify the factors influencing infection rates, a beta-binomial generalized linear mixed model (GLMM) was fitted, including species, cell type, and viral strain as fixed effects. The significance of these factors was assessed using analysis of deviance (Wald *χ*² tests, Table S5).

**Fig 1:**
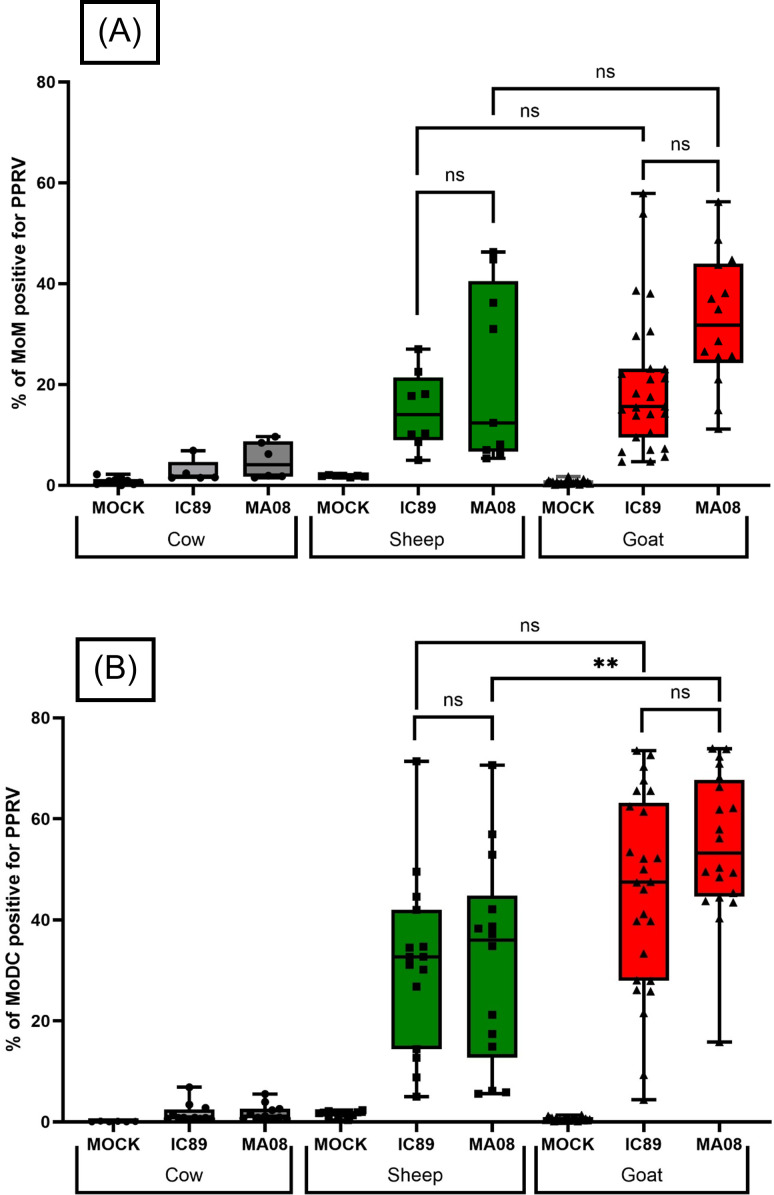
Percentage of MoMs (**A**) and MoDCs (**B**) from cow, sheep, and goats, positive for PPRV at 48 h post-infection (hpi) following *in vitro* infection with IC89 or MA08 strain. The MOCK group corresponds to uninfected control cells. Data are shown as individual biological replicates from a minimum of three animals per species and per condition, with boxplots representing the median and interquartile range; ns = non-significant; **P < 0.01. Statistical analysis was performed using a beta-binomial generalized linear mixed-effects model (GLMM). Post hoc comparisons were conducted using estimated marginal means (emmeans) with Tukey adjustment for multiple comparisons (see Tables S1 to S3).

**Fig 2:**
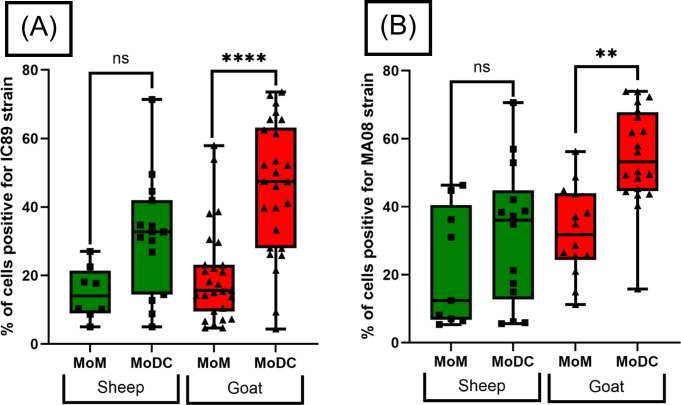
Percentage of MoMs and MoDCs from sheep and goats, positive for PPRV at 48 h post-infection (hpi) following *in vitro* infection with IC89 (**A**) or MA08 (**B**) strain. Data are shown as individual biological replicates from a minimum of three animals per species and per condition, with boxplots representing the median and interquartile range; ns = non-significant; **P < 0.01; ****P < 0.0001. Statistical analysis was performed using a beta-binomial generalized linear mixed-effects model (GLMM). Post hoc comparisons were conducted using estimated marginal means (emmeans) with Tukey adjustment for multiple comparisons (see Tables S1 and S4).

In goat MoMs, an average of 20% of the cells were positive for the N protein of PPRV strain IC89, compared with an average of 32.6% for those infected with strain MA08. Although no significant difference was found between the two strains (Fig. 1A; Table S2), a trend toward a lower proportion of infection with IC89 relative to MA08 was noted. In addition, in goat MoDCs, no significant difference was observed between the two viral strains, and in most cases, the cells were positive for NPPRV with a range of 40–73% (Fig. 1B; Table S2). Intercellular comparison (i.e., MoMs vs MoDCs) revealed a significant difference in the rate of NPPRV-positive cells, with MoDCs showing a significantly higher proportion of infection than MoMs for both PPRV strains (Fig. 2A and B; Table S3; odds ratios [ORs] > 2; *P* < 0.0001 for IC89 and OR = 1.94; *P* = 0.0057 for MA08).

For sheep MoMs, there was no significant difference in the rate of NPPRV-positive cells between the two PPRV strains, and the variability of responses was high, with an average rate of 14.7% for IC89 and 21% for MA08 (Fig. 1A; Table S2). Similarly, for sheep MoDCs, there was no significant difference between these two viral strains in terms of percentage of NPPRV-positive cells. In both cases, the average was 30%, with comparable extreme values (Fig. 1B; Table S2). Although MoDCs showed higher proportions of infection than MoMs (OR > 1), this difference was not statistically significant for either IC89 or MA08 (Fig. 2A and B; Table S3).

MoMs and MoDCs from cattle were also infected with IC89 and MA08 strains using the same parameters. Flow cytometry analysis after intracellular staining of the NPPRV protein revealed very low levels of NPPRV-positive cells across both cell types and viral strains (Fig. 1A and B). These infection levels were significantly lower than those observed for sheep and goats (OR < 1; *P* < 0.001; Table S4).

Inter-species comparisons further indicated that goats exhibited significantly higher proportions of NPPRV-positive cells than sheep in MoDCs for the MA08 strain (OR > 2; *P* = 0.0016; Table S4), while only a non-significant trend was observed for the IC89 strain (OR = 1.83; *P* = 0.058; Table S4). No significant differences were observed between the two species in MoMs (Table S4).

### Replication of PPRV in MoMs and MoDCs

To study viral replication in MoMs and MoDCs of different hosts, post-infection supernatants were added to cultures of SLAM-expressing Vero cells (VDS) (26) at dilutions from 10^−1^ to 10^−7^ with a minimum of four replicates per dilution. The cytopathic effect (CPE) was observed under the microscope, and viral titers were calculated as described in Materials and Methods.

To identify the factors influencing viral production, a negative binomial generalized linear mixed model was fitted, including species, cell type, and viral strain as fixed effects. The significance of these factors was assessed using analysis of deviance (Wald *χ*² tests, Table S5).

**Fig 3:**
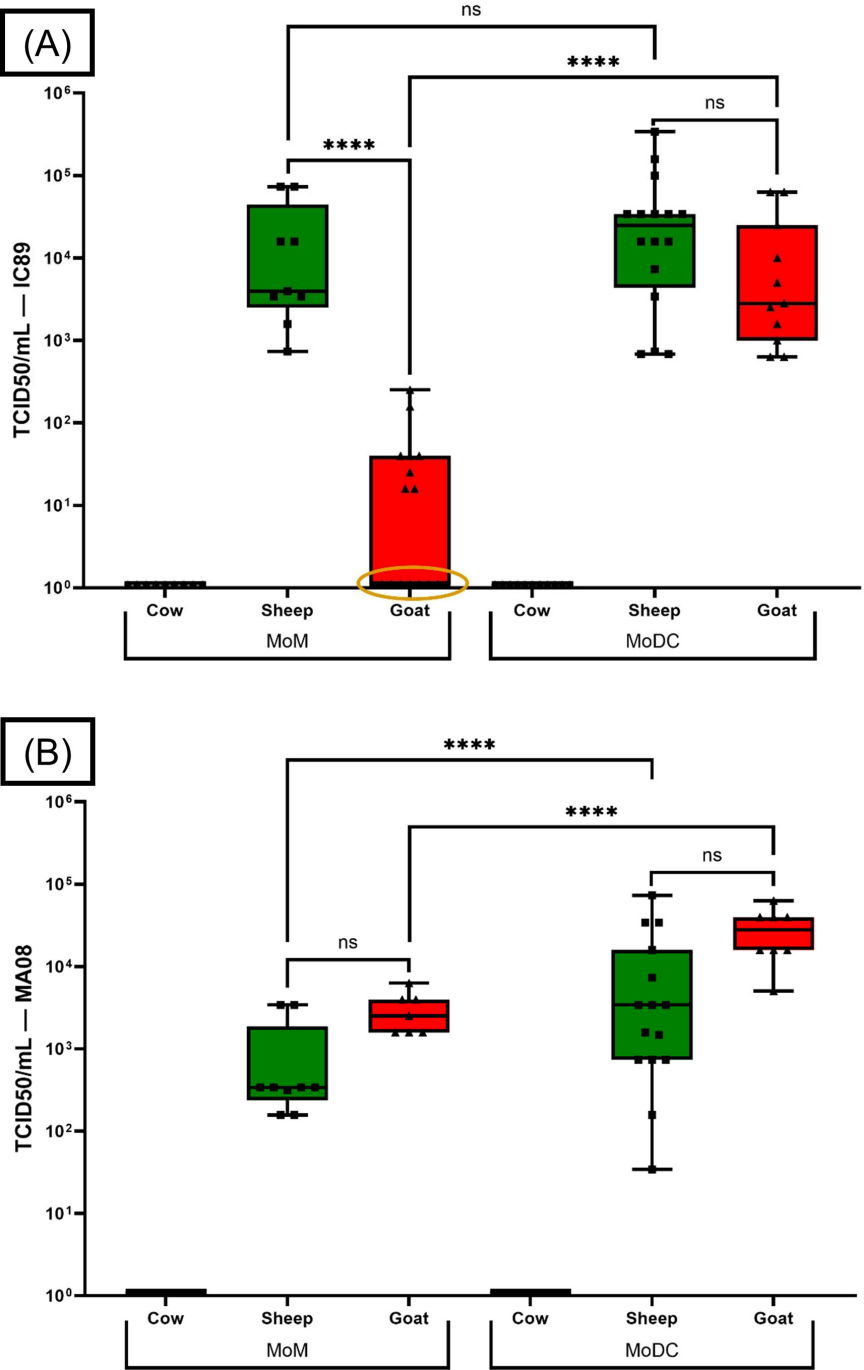
Viral titers (TCID_50_/mL) of IC89 (**A**) and MA08 (**B**) at 48 h post-infection (hpi) in cow, sheep, and goat MoMs and MoDCs. Nine of the seventeen replicates were below the limit of detection in goat MoMs infected with IC89 (circled in panel A). Data are shown as individual biological replicates from a minimum of three animals per species and per condition, with boxplots representing the median and interquartile range; ns = non-significant; ****P < 0.0001. Statistical analysis was performed on viral titers using a generalized linear mixed model (GLMM) fitted with a negative binomial distribution. Post hoc comparisons were performed using estimated marginal means with Tukey adjustment for multiple comparisons (see Tables S5 to S8).

In goats, post-infection supernatants from MoDCs had, on average for both PPRV strains MA08 and IC89, significantly higher titers than supernatants from MoMs (*P* < 0.0001; Fig. 3A and B; Table S6). In MoMs, viral titers in post-infection supernatants were significantly higher for the MA08 strain than for the IC89 strain (*P* < 0.0001; Table S7). Indeed, MA08 reached an average of 3 × 10^3^ TCID_50_/mL (Fig. 3B), whereas for IC89, viral titers were barely detectable, with an average of 34 TCID_50_/mL (Fig. 3A), including 9 out of 17 replicates in which no titer was detected (circled in Fig. 3A). In contrast, no significant differences were observed between the two strains in MoDCs (Table S7).

In sheep, viral titers were significantly higher for the MA08 strain in MoDCs than in MoMs (Ratio [*R*] > 9; *P* < 0.0001; Fig. 3B; Table S6), with a TCID_50_ of approximately 1.2 × 10^4^ for MoDCs compared to 9.8 × 10^2^ for MoMs. For the IC89 strain, viral titers tended to be higher in MoDCs than in MoMs (*R* > 2; *P* = 0.063; Fig. 3A, Table S6), although the difference did not reach statistical significance. The IC89 strain yielded a significantly higher mean titer than the MA08 strain in both MoDCs (*P* < 0.001; Fig. S1; Table S7) and MoMs (*P* < 0.0001; Fig. S1; Table S7).

The titration of 48 hpi supernatants of the IC89 strain on sheep MoMs versus goat MoMs indicated a significant difference (*P* < 0.0001; Table S8), with sheep MoMs exhibiting high titers with an average TCID_50_ of 2 × 10^4^. In contrast, goat MoMs infected with IC89 exhibited very low or undetectable viral titers, as described above (Fig. 3A). In cattle APCs, no viral titer was detected in the post-infection supernatants (Fig. 3A and B).

### Kinetics of infection of goat MoMs by PPRV IC89 strain

#### Infection and replication

After observing low or non-detectable viral titers in supernatants of goat MoMs infected for 48 h with the IC89 strain, infection kinetics were studied to see if replication was somewhat delayed for this low virulent strain. Goat MoMs were infected for 24, 48, 72, and 96 h, at an MOI of 0.1. As previously, the rate of NPPRV-positive cells was measured using a flow cytometer, and post-infection supernatants were collected, and viral titers were determined.

**Fig 4:**
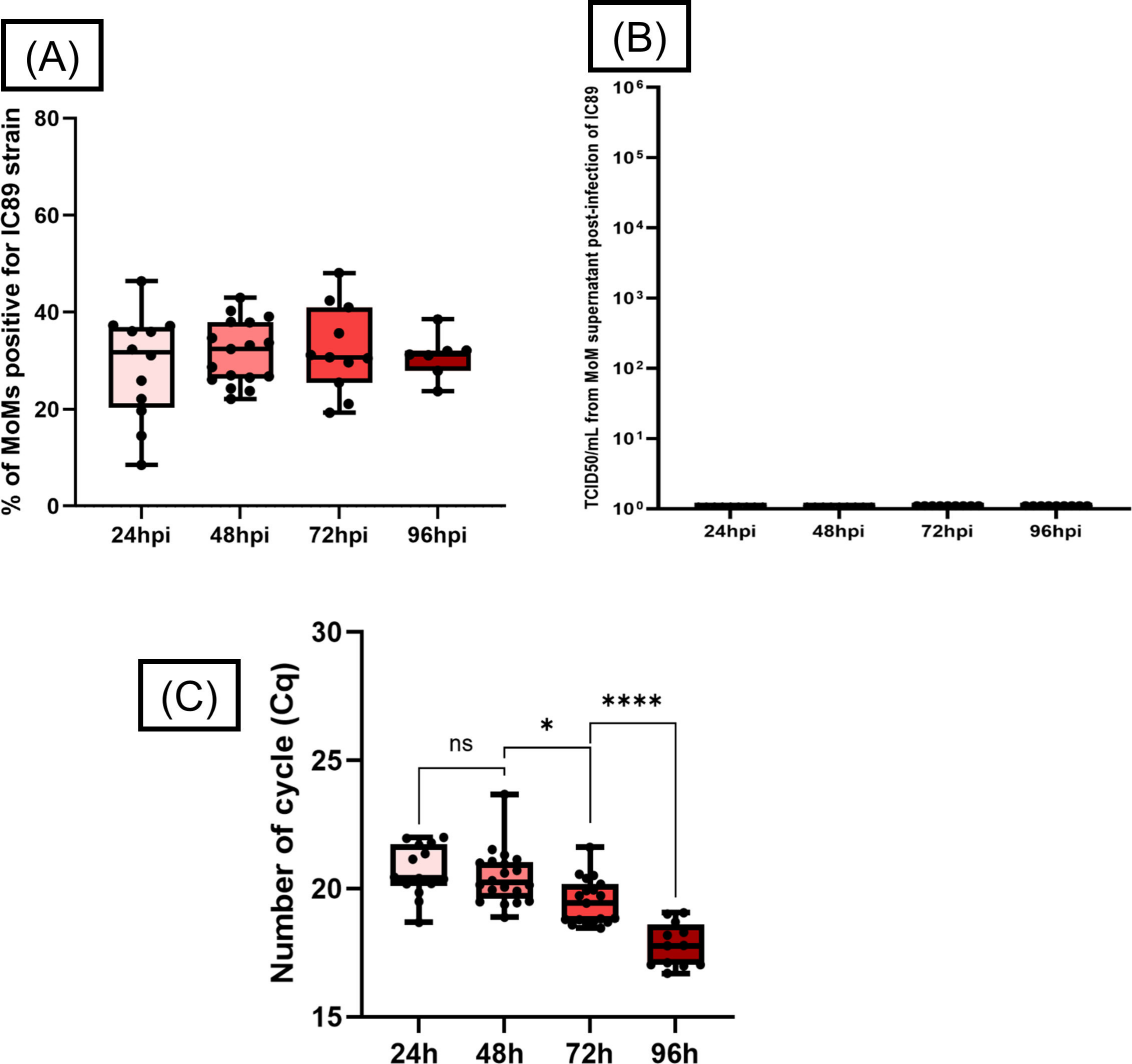
(**A**) Kinetics of infection of goat MoMs by the PPRV IC89 strain. Percentages of NPPRV+ cells were determined by flow cytometry at 24, 48, 72, and 96 h post-infection (hpi). Data are shown as individual biological replicates from three goats (minimum *n* = 7 replicates per point), with boxplots representing the median and interquartile range. A beta-binomial generalized linear mixed model (GLMM) revealed no significant effect of time post-infection on infection rates (all *P* > 0.76). Post hoc pairwise comparisons using Tukey-adjusted estimated marginal means (emmeans) confirmed the absence of significant differences between 24, 48, 72, and 96 hpi (all adjusted *P* > 0.99; see Table S9). (**B**) Viral titration kinetics of MoMs supernatants from goats infected with strain IC89 at 24, 48, 72, and 96 hpi. Data are shown as individual biological replicates from three goats with *n* = 9 replicates per time point. (**C**) Kinetics of viral RNA in supernatants of goat MoMs infected with the IC89 strain at 24, 48, 72, and 96 hpi. Viral RNA levels were quantified by RT-qPCR (expressed as Cq values) targeting the PPRV N gene. Each point represents an individual biological replicate from three goats, with *n* ranging from 12 to 20 replicates per time point, with boxplots representing the median and interquartile range. Statistical analysis was performed using a linear mixed-effects model (LMEM) fitted by restricted maximum likelihood (REML). Pairwise comparisons between time points were conducted using estimated marginal means (emmeans) with Tukey adjustment for multiple comparisons (Kenward–Roger approximation for degrees of freedom). Corresponding *P* values are reported in Table S10. Statistically significant differences are indicated as: ns = non-significant; *P < 0.05; ****P < 0.0001.

The results show that the rate of infected cells is relatively stable over time, with an average NPPRV positivity of around 30% regardless of the infection time. No significant increase in the percentage of NPPRV-positive cells was observed between 24 and 96 hpi (Fig. 4A; Table S9).

The post-infection supernatants from IC89-infected goat MoMs were collected in triplicates per animal throughout the kinetics and titrated on VDS. No TCID_50_/mL viral titers could be detected for any time point during the kinetics, with values remaining below the test detection threshold (Fig. 4B), and cytopathic effects (CPEs) sometimes observed in a single well of the first dilution (10^−1^). The titration plates used for this kinetic analysis were then labeled with the monoclonal antibody 38.4 targeting the NPPRV, coupled with tetramethylrhodamine isothiocyanate (TRITC), in order to observe the presence or absence of the virus at the cellular level. We observed the presence of the virus in VDS cells in the first dilution, and sometimes up to the second and third dilutions (10^−2^ and 10^−3^). However, this detection occurred in the absence of a cytopathic effect (CPE), or with CPE limited to a single well in the first dilution, which is insufficient to calculate a reliable viral titer (Fig. S2).

#### Detection of viral RNA

To determine the presence of viral genomic RNA, an RT-qPCR was performed on post-infection supernatants throughout the kinetic. The cycle number (Cq) between 24 and 48 hpi remained stable at around 20.5. A decrease in the Cq value was first observed at 72 hpi, falling to an average of 19.5, with an even more significant decrease at 96 hpi, when the Cq averaged 17.8 (*P* < 0.0001; Fig. 4C; Table S10), representing an increase in genetic material of the IC89 strain detected in supernatants.

### Detection of dsRNA and NPPRV protein in goat MoMs and MoDCs

**Fig 5:**
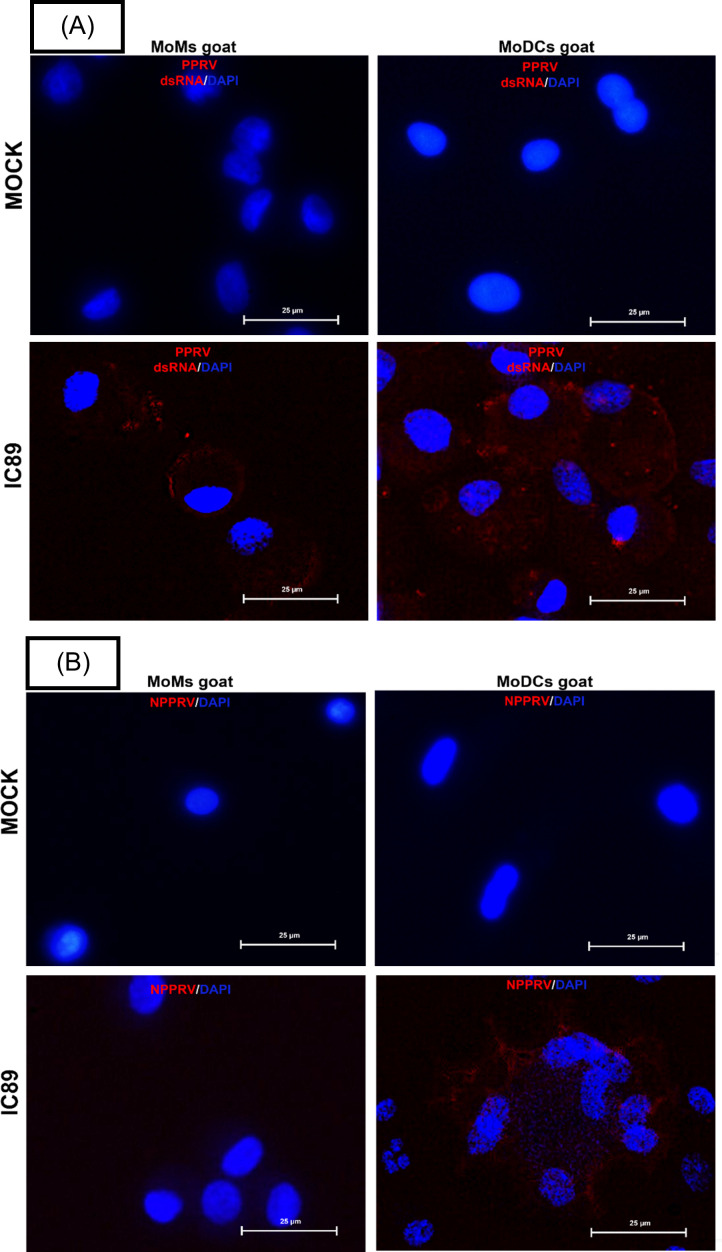
Immunofluorescent detection of viral double-stranded RNA (dsRNA) (**A**) and nucleoprotein PPRV protein (NPPRV) (**B**) in goat MoMs and MoDCs infected with IC89. Infected and uninfected (MOCK) MoMs and MoDCs were stained with DAPI to identify cell nuclei, dsRNA (**A**), and NPPRV (**B**) are detected here in red, scale bar = 25 µm.

To investigate a possible blockage or slowdown in the replication cycle of the IC89 strain in goat MoMs, immunofluorescence was used to detect separately double-stranded RNA (dsRNA), an indicator of the accumulation of aberrant viral RNAs in morbilliviruses, and the PPRV nucleoprotein (NPPRV), a marker of viral protein expression, in infected MoMs. The profiles obtained were compared with those observed in goat MoDCs. A positive dsRNA signal was observed in both cell types in the presence of IC89, although the dsRNA signal appeared slightly less intense in MoMs (Fig. 5A). The dsRNA signal appeared stronger in goat MoMs when infected by MA08 (Fig. S3). NPPRV is detected in goat MoDCs infected with IC89, but not in goat MoMs (Fig. 5B).

## DISCUSSION

PPRV infection can lead to a variety of outcomes ranging from acute symptoms and death of the infected host to subclinical infection. Surveillance and control measures must be adapted to provide an efficient response. However, we have limited capacity to predict disease outcomes in different hosts and prepare accordingly. A better understanding of differences in sensitivity of the host to viral strains, studied *in vitro*, would make it possible to anticipate disease impacts and develop a predictive model that can demonstrate not only the sensitivity of a host to different PPRV strains but also the differences in virulence expressed by the same strain depending on the host (27, 28). To move toward this objective, we selected two strains of PPRV with distinct virulence profiles in goats, mildly virulent IC89 and highly virulent MA08 (24, 29, 30). Goat and sheep cells were used because these species are the main natural hosts of PPRV, while bovine cells served as a relevant negative control, as cattle are considered a dead-end host (20, 31). This *in vitro* experimental model focused on a comparative overview of PPRV infection in MoMs and MoDCs to investigate differences in sensitivity and replication profile between cell types, host species, and across viral strains.

Overall, our study shows that MoDCs from goats and sheep are more permissive to PPRV than MoMs. On average, the rate of infected cells (or NPPRV-positive cells) and viral titers is higher for MoDCs than for MoMs. In cattle, the absence of infection and of measurable replication in MoDCs and MoMs confirms the dead-end host status of this species.

Despite significant inter-individual variability observed in the number of infected cells during the experiment, the measurement of viral replication via titration provided a robust and biologically meaningful reading for defining permissiveness. The data obtained 48 h post-infection at an MOI of 0.1 likely reflect the combined effect of several infectious cycles, incorporating both the efficiency of viral replication and the virus’s capacity for secondary spread in cell cultures. Experiments at lower MOI and at different times post-infection may give additional information on the PPRV primary infectious cycle in both cells; however, our results show that the conditions used in our *in vitro* experiments are suitable to detect differences of interest for the overall goal of this study.

No significant differences in viral titers were observed between the IC89 and MA08 viral strains in goat MoDCs. However, in sheep MoDCs, viral titers were significantly higher for the IC89 strain than for the MA08 strain. MoDCs results concur with the multiple field and experimental studies showing that cattle are not affected and cannot transmit PPRV, unlike the main small ruminant hosts (20, 32). This cell model could therefore potentially serve as an indicator of the ability of the virus to infect a given species. In small ruminants, MoDCs confirmed their susceptibility to PPRV infection but did not reveal consistent differences between goats and sheep, with a significant difference observed for MA08 but only a non-significant trend for IC89. Additional experiments with other PPRV strains and with MoDCs of other small ruminant breeds are needed to explore potential differences in the susceptibility of MoDCs of sheep and goats to PPRV infection.

In contrast to MoDCs, MoMs are less permissive but more discriminating host cells for PPRV infection. In goats, strain MA08 infected MoMs significantly more efficiently than strain IC89. Conversely, IC89 produced detectable titers that were significantly higher than MA08 in sheep MoMs. Furthermore, the titers observed with IC89 in sheep MoMs were significantly higher than those obtained in goat MoMs. This suggests that the ability of this strain to replicate depends on both host species and the infected cell type. Based on this, and on knowledge of the virulence of these strains *in vivo* (21, 24, 29, 30, 33), we propose that this infection model with MoMs could serve as an indicator of strain-specific virulence in small ruminant breeds and may highlight host-specific signatures of viral adaptation.

For the remainder of the study, infection kinetics were performed on goat MoMs infected with IC89, since the discrepancy was most pronounced in this model (i.e., cellular infection was detected by flow cytometry, but the viral titer was undetectable). The objective was to determine whether the absence of a viral titer was due to a delay in replication or a persistent blockage of the viral cycle. Throughout the kinetics (24–96 h post-infection), the rate of infected cells remained stable, averaging between 28% and 32% with no significant increase, suggesting continuous infection of a sub-population of macrophages present in the culture. Titrations of supernatants at all time points remained negative, indicating the absence of measurable infectious virion production. Nevertheless, RT-qPCR revealed a significant decrease in Cq between 48, 72, and 96 hpi, reflecting a gradual accumulation of extracellular viral genomic RNA. It should be noted that RT-qPCR does not distinguish between infectious and non-infectious viral particles. In addition, immunofluorescence microscopy of the titration plates showed that viral antigens were present in the cells, as shown by NPPRV protein detection in VDS cells, at different levels but without CPE, which is consistent with the absence of measurable viral titer and the cytometry results.

Despite the absence of measurable infectious titer, the detection of viral RNA in the supernatants suggests an incomplete or an abortive replication cycle. This could result in the release of non-infectious or defective viral particles, which has already been observed with several RNA viruses (34–36), including morbilliviruses (37–40).

In negative-sense single-stranded RNA viruses, such as PPRV, viral genome replication is closely linked to nucleocapsid assembly, meaning that dsRNA is not considered a reliable indicator of productive replication (41). Nevertheless, the presence of dsRNA is more likely to be an indicator of the accumulation of aberrant viral RNA, such as defective interfering RNA (DIs) or defective viral genomes (DVGs) (39). Such DIs and DVGs can be produced during virus production, but the contrasting levels of dsRNA observed in MoMs and MoDCs infected with IC89 suggest differences in the accumulation of these particles during infection. Still, it would be interesting to evaluate the amounts of DIs and DVGs commonly accumulating in PPR virus productions, as it may have impacted in some ways many *in vivo* and *in vitro* experimental studies on PPRV pathogenesis and host immune responses published so far.

Importantly, no NPPRV signal was detected in MoMs infected with the IC89 strain, unlike in MoDCs. This would suggest that the IC89 viral cycle in MoMs appears to be engaged until the accumulation of aberrant viral RNA forms (DIs or DVGs), while nucleoprotein expression or accumulation is severely limited or absent. Therefore, while partial expression of the viral genome is detectable, this does not result in the production of infectious particles, indicating a restricted or semi-permissive infection of goat MoMs by the IC89 strain. Such a restriction may be associated with the activation of interferon-dependent antiviral pathways known to limit viral protein synthesis (42, 43). This result is unlikely to be due to the presence of aberrant viral RNA forms in virus stocks used, as these stocks have led to successful infection with IC89 and MA08 strains in all sheep and goat APCs except goat MoMs for IC89 strain, and in previous *in vitro* and *in vivo* experimental studies performed with similar IC89 and MA08 virus productions (24, 29, 30).

Nevertheless, further work is needed to precisely understand the observations made within this promising model in order to evaluate its usefulness. It would be interesting to move toward a multiomics approach to understand the different mechanisms involved in PPRV infection of MoMs and MoDCs, in particular those related to innate antiviral immune activity and the link between different immune signaling pathways and the non-structural proteins C and V of morbilliviruses. It would also be relevant to quantify the different production of cytokines in the post-infection supernatants of MoMs and MoDCs, and to perform proteomic analysis of these supernatants to detect and characterize viral proteins released during infection. Finally, observing the state of the virion as it leaves the cells under electron microscope would provide additional structural information on the viral particles produced and their infectious capacity. Future studies should explore the potential impact of aberrant viral RNA forms present in virus stocks on the models used to understand PPRV pathogenesis and host immune response.

In summary, here we have highlighted the difference in permissiveness to PPR virus strains between MoDCs and MoMs. As demonstrated by the results we obtained with the IC89 and MA08 strains in the two cell models obtained from three different hosts (goats, sheep, and cattle), *in vitro* infections of MoDCs may enable us to determine whether a given host is susceptible to the PPR virus, and MoMs could serve as a cell model to determine the virulence of a given strain among susceptible hosts. With the IC89 strain, an abortive infection is observed in goat MoMs, while sheep MoMs are completely permissive. MoMs from goat or sheep also remain permissive for the MA08 strain. The use of bovine MoMs and MoDCs in this study constitutes a relevant negative control due to its status as a dead-end host. This combined model of two cell types (MoDCs and MoMs) would be useful for pre-screening studies to assess the sensitivity of species to a given strain of PPRV and thus estimate relative virulence. Testing other PPRV strains with goat, sheep, and cattle MoMs and MoDCs, and extending such tests to other host species will confirm the utility of this model.

Since 2024, Europe has been facing the emergence of PPR, with small ruminants infected in seven different countries and showing a wide variety of symptoms (3, 44–46). This recent dynamic in the spread of PPRV shows that the epidemiological situation remains unstable and that the virus still crosses borders, making it essential to strengthen risk assessment tools and early detection capacities. The risk is also present in areas that interface with wildlife, with cases observed in atypical hosts and endangered wild ruminants in contact with domestic animals (47–51). Furthermore, the impact of PPRV on wild species remains poorly understood, as does their potential role in the transmission or maintenance of the virus and modulation of virulence. As global eradication efforts led by FAO and WOAH intensify, having tools to anticipate host susceptibility and strain virulence would be a major asset in guiding early detection and rapid response measures.

## MATERIALS AND METHODS

### Cell lines and viral production

All viral strains were produced from Vero expressing the canine SLAM receptor (or Vero-Dog-SLAM cells, VDS cells) (26). VDS cells were cultured in complete Dulbecco’s modified Eagle’s medium (DMEM, Gibco) supplemented with 10% fetal bovine serum (FBS, Dominique Dutscher). Virus stocks were harvested from infected cell supernatants, clarified by centrifugation, and stored at −80°C until use. The PPRV strains IC89 (lineage I) and MA08 (lineage IV) are from productions used in previous experimental studies (21). Any risk of genetic drift of PPRV strains during cell passages was assessed by sequencing the full genome of the virus production used for the infection experiments using a protocol described elsewhere (52). Genomic comparison showed that no mutations were detected following the passages required for production.

### Differentiation of macrophages and dendritic cells from CD14^+^ monocytes

Peripheral blood samples were obtained from clinically healthy goats (*Capra hircus*, *N* = 3), sheep (*Ovis aries*, *N* = 3), and cattle (*Bos taurus*, *N* = 6) housed at the CIRAD animal facility (Montpellier, France).

Peripheral blood mononuclear cells (PBMCs) were isolated from the whole blood of the animals studied by Ficoll-Hypaque density gradient centrifugation using Histopaque-1077 (Sigma-Aldrich), following standard protocols (53). After collection, viable PBMCs were counted in a hemocytometer using the Trypan Blue exclusion method.

CD14^+^ monocytes were positively selected using CD14 MicroBeads UltraPure (Miltenyi Biotec) and LS MACS columns, according to the manufacturer’s instructions. After elution, CD14^+^ monocytes were cultured in Roswell Park Memorial Institute medium and GlutaMAX (RPMI 1640 + GlutaMAX, Gibco) supplemented with 10% FBS and 1% antibiotics (Penicillin-Streptomycin). A minimum purity of 80% CD14^+^ monocytes is required to ensure homogeneous differentiation into MoMs or MoDCs (Fig. S4).

For differentiation into monocyte-derived dendritic cells (MoDCs), cultures were supplemented with in-house bovine recombinant IL-4 and GM-CSF (50 ng/mL). From plasmid prepared with EndoFree Plasmid Purification Maxi Kit Qiagen (reference 12362), cytokines were then produced by transfection on HEK 293T cells. For the differentiation into monocyte-derived macrophages (MoMs), no exogenous growth factors were added, and monocytes were allowed to spontaneously differentiate into macrophages under standard culture conditions. This approach, although less controlled than cytokine-supplemented protocols, mimics a more physiological environment and results in a heterogeneous macrophage population (54, 55). Differentiation was carried out for 6–7 days with a medium change on day 2 or 3 (Fig. S5). In goats, characterization based on CD205 marker expression was performed to distinguish monocytes from cells differentiated into MoMs or MoDCs (Fig. S6A). Similarly, the CD209 marker was used in cattle (56) (Fig. S6B). To distinguish MoMs from MoDCs in goats and sheep, the CD172a marker was used (Fig. S6C). In addition, SLAMF9 receptor (homolog of SLAMF1) labeling was also performed on goat cells (Fig. S6D). Viral infection assays were performed at the end of the differentiation period. In addition to phenotyping, microscopic observation (Fig. S5) confirmed that the cells had been differentiated in agreement with previous work (15, 56, 57).

### Viral infection

MoDCs and MoMs were infected with either MA08 or IC89 strains at a multiplicity of infection (MOI) of 0.1. The cells were incubated with virus for 1 h at 37°C in serum-free RPMI. After incubation, the cells were washed with PBS and cultured for 48 h in RPMI supplemented with 2% FBS. Forty-eight hours post-infection, the culture supernatants were harvested, aliquoted, and stored at –80°C for downstream analysis.

### Flow cytometry

Following infection, adherent cells in culture were detached using TrypLE Express Enzyme 1× (Gibco) at 37°C for 10 min. The cells were harvested into PBS, centrifuged at 300 × *g*) for 5 min at 4°C, and fixed with 4% paraformaldehyde (or 4% PFA) for 10 min at 4°C.

For intracellular PPRV detection, the cells were permeabilized with 0.3% saponin and stained with FITC-conjugated monoclonal antibody (clone 38.4) targeting the N nucleoprotein of PPRV developed by Libeau and Lefevre (58) and produced by ProteoGenix (Schiltigheim, France). No secondary antibody was required due to conjugation of Mab 38.4 with FITC. Stained cells were analyzed using a BD FACSCanto II flow cytometer (BD Biosciences) and FlowJo_V10 software (Fig. S7).

For phenotyping, the primary antibodies anti-CD14 (CAM36A), anti-CD205 (ILA114A), anti-CD209 (209MD26A), anti-CD172a (DH59B), and anti-SLAMF9 CACT206A were provided by WSU Monoclonal Antibody Center Veterinary Microbiology and Pathology (Washington State University – Pullman). The secondary antibodies used were rat anti-mouse IgG1, eBioscience, PE-Cyanine7 Clone M1-14D12 (reference: 24-4015-82), Invitrogen by Thermo Fisher Scientific, and Alexa Fluor goat anti-mouse IgG2a (reference: A21241), Invitrogen by Thermo Fisher Scientific.

### Titration of PPRV

Viral production and cell culture supernatants were titrated on VDS cells seeded in 96-well plates using the Spearman-Kärber method (59–62). Infectious titers were calculated as TCID_50_/mL using the TCID_50_ calculator developed by Marco Binder (v2.1-20-01-2017_MB* by Marco Binder; adapted @ TWC https://www.klinikum.uni-heidelberg.de/fileadmin/inst_hygiene/molekulare_virologie/Downloads/TCID50_calculator_v2_17-01-20_MB.xlsx). The titration plates were marked with antibody 38:4 (anti-NPPRV) coupled with TRITC to observe the presence or absence of the virus (Fig. S2), according to the Eloiflin et al. (63) protocol.

### RNA extraction from supernatant and real-time qPCR analyses

Viral RNA was extracted from supernatants using the IndiMag Pathogen Kit (Indical Biosciences), following the manufacturer’s protocol. RNA isolation was performed on the IDEAL 32 automated extraction system (Innovative Diagnostics), based on magnetic bead technology.

RNA of PPRV present in the supernatants was quantified using real-time PCR by partial amplification of the N protein gene using the ID Gene peste des petits ruminants Duplex kit (Innovative Diagnostics) and then performed with LightCycler (Roche).

### Immunofluorescence staining and inverted microscopy

The cells were fixed at 48 hpi using 4% paraformaldehyde (PFA) for 20 min at room temperature. After fixation, permeabilization was performed with 0.1% Triton X-100 in 1× PBS for 5 min at room temperature. The cells were then incubated in a buffer composed of 1× PBS, 10% FBS, and 1 g BSA per 50 mL for 30 min. Primary staining was performed with anti-dsRNA monoclonal antibody J2 (IgG2a, 1 mg/mL, SCICONS, Jena Bioscience), diluted 1:300, and incubated for 2 h at room temperature. For detection, the cells were incubated with DAPI diluted 1:1,000 (Sigma-Aldrich, MBD0015) and either a Donkey anti-Mouse IgG secondary antibody (H + L) Alexa Fluor 555 secondary antibody (2 mg/mL) applied at 4 µg/mL (Thermo Fisher Scientific, Cat# A-31570, RRID:AB_2536180), or with TRITC-coupled anti-NPPRV (Mab 38.4) antibody diluted 1:250. In both cases, incubation took place for 1 h at room temperature, in the dark. The samples were then imaged using an AXIOVERT A1 inverted microscope (Zeiss, France) with Archimed 6.1.4 software, and analyzed with ImageJ (version supplied with Java 1.8.0_172 64-bit).

### Statistical analysis

Statistical analyses were performed, and graphs were generated using GraphPad Prism v.10.6.1 and R software v.4.5.2. For the analysis of infection rates, generalized linear mixed models (GLMMs) were fitted using the *glmmTMB* package, with a beta-binomial distribution to account for overdispersion in proportional data (CellPos/CellNeg obtained by flow cytometry). Species, cell type, and viral strain were included as fixed effects, and a nested random effect (1| ID/Sample) was included to account for repeated measurements within individuals, where ID represents the animal identity, and Sample represents the experimental sample. The initial model included all possible interactions between species, cell type, and viral strain. Model simplification was then performed by removing non-significant higher-order interactions to obtain a simpler and more interpretable final model, while retaining biologically relevant terms.

Viral titers (TCID_50_) were analyzed using GLMM with a negative binomial distribution (*nbinom2*), also fitted using *glmmTMB*. Species, cell type, and viral strain were included as fixed effects, with the same nested random structure (1| ID/Sample).

For kinetic experiments, the analysis was performed using generalized linear mixed models (GLMMs) with a beta-binomial distribution in cases of overdispersion, with post-infection time (hpi) as a fixed effect, with the same nested random structure (1| ID/Sample).

RT-qPCR data from kinetics were analyzed using linear mixed-effects models (LMEMs) fitted by restricted maximum likelihood (REML) method, with hpi as a fixed effect and animal identity as a random effect to account for repeated measurements in the same animal. Degrees of freedom were estimated using the Kenward–Roger approximation.

Analysis of deviance was performed using Wald *χ*² tests to assess the significance of fixed effects. Pairwise comparisons were conducted using estimated marginal means (emmeans) with Tukey adjustment for multiple testing. Model diagnostics were performed using the DHARMa package, including assessment of residual distribution and dispersion.

## Data Availability

The data that support the findings of this study are available at https://doi.org/10.18167/DVN1/SHFVNB.

## SUPPLEMENTAL MATERIAL

Supplemental figures jvi.00294-26-s0001.docx Fig. S1 to S7.

Supplemental tables jvi.00294-26-s0002.docx Tables S1 to S10.

ASM does not own the copyrights to Supplemental Material that may be linked to, or accessed through, an article. The authors have granted ASM a non-exclusive, world-wide license to publish the Supplemental Material files. Please contact the corresponding author directly for reuse.
