# Supplementary material for: Strain-dependent differences in the capacity of peste des petits ruminants virus to infect antigen-presenting cells
Source: J Virol. 2026 May 27;100(6):e00294-26. doi: 10.1128/jvi.00294-26 (PMC13288782; doi:10.1128/jvi.00294-26)
Supplement: Supplemental tables — Tables S1 to S10. [file jvi.00294-26-s0002.docx]

**Table S1: Analysis of deviance of a beta-binomial generalized linear mixed model (GLMM) using χ² tests.** The response variable was the proportion of positive versus negative cells (cbind(CellPos, CellNeg)). χ² corresponds to Wald chi-square statistic and Df to degrees of freedom. Random effects were included as (1 | ID/Sample). Statistically significant effects (P < 0.05) are highlighted in grey. Negative controls (MOCK) were excluded from the analysis due to the expected absence of infection.

| **Effect** | **Χ^2^** | **Df** | **p-value** |
| --- | --- | --- | --- |
| Species | 69.98 | 2 | <0.0001 |
| Cell type | 65.07 | 1 | <0.0001 |
| Strain | 2.05 | 1 | 0.152 |
| Species x Cell type | 11.20 | 2 | 0.0037 |
| Species x Strain | 0.41 | 2 | 0.815 |
| Cell type x Strain | 0.77 | 1 | 0.380 |
| Species x Cell type x Strain | 0.55 | 2 | 0.761 |

**Table S2: Pairwise comparisons of infection proportion between species using a beta-binomial generalized linear mixed model (GLMM) and an estimated marginal means (emmeans).** Odds ratios (OR) compare the proportion of infected cells between species for each cell type and viral strain. OR < 1 indicates a lower proportion of infection in the first species listed in the comparison. P values are from Wald z-tests and adjusted using the Tukey method for multiple comparisons. OR are presented with 95% confidence intervals (CI). Statistically significant results (P < 0.05) are highlighted in grey.

| **Cell type** | **Strain** | **Comparison** | **Odds ratio (95% CI)** | **p-value** |
| --- | --- | --- | --- | --- |
| MoDCs | IC89 | Cow/Goat | 0.042 (0.015–0.117) | <0.0001 |
| MoDCs | IC89 | Cow/Sheep | 0.100 (0.035–0.29) | <0.0001 |
| MoDCs | IC89 | Goat/Sheep | 2.40 (1.24–4.65) | 0.0055 |
| MoMs | IC89 | Cow/Goat | 0.121 (0.038–0.392) | <0.0001 |
| MoMs | IC89 | Cow/Sheep | 0.137 (0.039–0.479) | 0.0006 |
| MoMs | IC89 | Goat/Sheep | 1.13 (0.51–2.51) | 0.93 |
| MoDCs | MA08 | Cow/Goat | 0.043 (0.016–0.116) | <0.0001 |
| MoDCs | MA08 | Cow/Sheep | 0.11 (0.039–0.291) | <0.0001 |
| MoDCs | MA08 | Goat/Sheep | 2.48 (1.25–4.93) | 0.0054 |
| MoMs | MA08 | Cow/Goat | 0.126 (0.042–0.378) | <0.0001 |
| MoMs | MA08 | Cow/Sheep | 0.203 (0.062–0.668) | 0.0049 |
| MoMs | MA08 | Goat/Sheep | 1.62 (0.68–3.82) | 0.39 |

**Table S3: Pairwise comparisons of infection proportion between viral strains estimated using a beta-binomial generalized linear mixed model (GLMM) and an estimated marginal means (emmeans).** Odds ratios (OR) compare the proportion of infected cells between viral strains within each species and cell type. OR < 1 indicates that the first strain listed has a lower proportion of infection compared to the second strain. P values are from Wald z-tests and adjusted using the Tukey method for multiple comparisons. OR are presented with 95% confidence intervals (CI).

| **Species** | **Cell type** | **Comparison** | **Odds ratio (95% CI)** | **p-value** |
| --- | --- | --- | --- | --- |
| Goat | MoDCs | IC89 / MA08 | 0.91 (0.65–1.26) | 0.57 |
| Goat | MoMs | IC89 / MA08 | 0.70 (0.47–1.04) | 0.079 |
| Sheep | MoDCs | IC89 / MA08 | 0.94 (0.63–1.40) | 0.76 |
| Sheep | MoMs | IC89 / MA08 | 1.0 (0.53–1.90) | 0.99 |

**Table S4: Pairwise comparisons of infection proportion between cell types estimated using a beta-binomial generalized linear mixed-model (GLMM) and estimated marginal means (emmeans).** Odds ratios (OR) compare the proportion of infected cells between MoDCs and MoMs within each species and viral strain. OR > 1 indicates a higher proportion of infection in MoDCs relative to MoMs. P values are from Wald z-tests and adjusted using the Tukey method for multiple comparisons. OR are presented with 95% confidence intervals (CI). Statistically significant results (P < 0.05) are highlighted in grey.

| **Species** | **Strain** | **Comparison** | **Odds ratio (95% CI)** | **p-value** |
| --- | --- | --- | --- | --- |
| Goat | IC89 | MoDCs/MoMs | 3.14 (2.33–4.22) | <0.0001 |
| Sheep | IC89 | MoDCs/MoMs | 1.47 (0.86–2.54) | 0.161 |
| Goat | MA08 | MoDCs/MoMs | 2.42 (1.64–3.57) | <0.0001 |
| Sheep | MA08 | MoDCs/MoMs | 1.58 (0.91–2.74) | 0.106 |

**Table S5: Analysis of deviance of the selected negative binomial generalized linear mixed model (nbinom2) for viral titers (TCID50).** Viral titer (TCID50) was modelled as a function of species, cell type, and strain, including all interactions. A nested random effect (1 | ID/Sample) was included to account for repeated measurements. χ² values correspond to Wald test statistics and Df indicates degrees of freedom. Significant effects (p < 0.05) are highlighted in grey. Negative controls (MOCK) and the bovine species, considered non-permissive, were excluded due to the expected absence of viral replication (structural zero values).

| **Effect** | **Χ^2^** | **Df** | **p-value** |
| --- | --- | --- | --- |
| Species | 2.43 | 1 | 0.12 |
| Cell type | 84.9 | 1 | <0.0001 |
| Strain | 6.33 | 1 | 0.012 |
| Species x Cell type | 9.44 | 1 | 0.002 |
| Species x Strain | 63.2 | 1 | <0.0001 |
| Cell type x Strain | 0.25 | 1 | 0.62 |
| Species x Cell type x Strain | 12.1 | 1 | <0.001 |

**Table S6: Pairwise comparisons of viral production (TCID₅₀) between cell types (MoDCs/MoMs) using a negative binomial generalized linear mixed model (GLMM) and estimated marginal means (emmeans).** Ratios represent fold changes in infectious titers (MoDCs relative to MoMs) within each species and viral strain. Ratios > 1 indicate higher viral production in MoDCs compared to MoMs. P values are based on Wald z-tests with Tukey adjustment for multiple comparisons. Ratios are presented with 95% confidence intervals. Statistically significant results (P < 0.05) are highlighted in grey.

| **Species** | **Strain** | **Comparison** | **Ratio (95% CI)** | **P value** |
| --- | --- | --- | --- | --- |
| Goat | IC89 | MoDCs/MoMs | 68.8 (24.8–191) | <0.0001 |
| Goat | MA08 | MoDCs/MoMs | 9.57 (3.51–26.1) | <0.0001 |
| Sheep | IC89 | MoDCs/MoMs | 2.78 (1.13–6.83) | 0.025 |
| Sheep | MA08 | MoDCs/MoMs | 9.62 (3.93–23.5) | <0.0001 |

**Table S7: Pairwise comparisons of viral production (TCID₅₀) between viral strains (IC89/MA08) using a negative binomial generalized linear mixed model (GLMM) and estimated marginal means (emmeans).** Ratios represent fold changes in infectious titers (IC89 relative to MA08) within each species and cell type. Ratios > 1 indicate higher viral production for IC89 compared to MA08, whereas ratios < 1 indicate lower viral production for IC89 relative to MA08. P values are based on Wald z-tests with Tukey adjustment for multiple comparisons. Ratios are presented with 95% confidence intervals. Statistically significant results (P < 0.05) are highlighted in grey.

| **Species** | **Cell type** | **Comparison** | **Ratio (95% CI)** | **p-value** |
| --- | --- | --- | --- | --- |
| Goat | MoDCs | IC89/MA08 | 0.53 (0.21–1.33) | 0.176 |
| Goat | MoMs | IC89/MA08 | 0.07 (0.03–0.20) | <0.0001 |
| Sheep | MoDCs | IC89/MA08 | 5.40 (2.40–12.2) | <0.0001 |
| Sheep | MoMs | IC89/MA08 | 18.7 (7.43–46.9) | <0.0001 |

**Table S8: Pairwise comparisons of viral production (TCID₅₀) between species (Goat/Sheep) using a negative binomial generalized linear mixed model (GLMM) and estimated marginal means (emmeans).** Ratios represent fold changes in infectious titers (goats relative to sheep) within each cell type and viral strain. Ratios > 1 indicate higher viral production in goats compared to sheep, whereas ratios < 1 indicate lower viral production in goats relative to sheep. P values are based on Wald z-tests with Tukey adjustment for multiple comparisons. Ratios are presented with 95% confidence intervals. Statistically significant results (P < 0.05) are highlighted in grey.

| **Cell type** | **Strain** | **Comparison** | **Ratio (95% CI)** | **p-value** |
| --- | --- | --- | --- | --- |
| MoDCs | IC89 | Goat/Sheep | 0.31 (0.09–1.13) | 0.075 |
| MoDCs | MA08 | Goat/Sheep | 3.17 (0.84–11.95) | 0.089 |
| MoMs | IC89 | Goat/Sheep | 0.013 (0.003–0.051) | <0.0001 |
| MoMs | MA08 | Goat/Sheep | 3.18 (0.77–13.1) | 0.11 |

**Table S9: Pairwise comparisons of infection kinetics in goat MoMs infected with the IC89 strain using a** **beta-binomial generalized linear mixed model model (GLMM) and estimated marginal means (emmeans).** Odds ratios (OR) represent changes in the proportion of infected cells between time points post-infection (hpi). Odds ratios > 1 indicate an increased probability of infection at the later time point, whereas values < 1 indicate a decreased probability. P values are based on Wald z-tests with Tukey adjustment for multiple comparisons. In all comparisons, odds ratios were close to 1 and not statistically significant, indicating no meaningful change in the probability of infection over time.

| **Comparison** | **Odds ratio** | **p-value** |
| --- | --- | --- |
| 24hpi vs 48hpi | 0.917 | 0.991 |
| 24hpi vs 72hpi | 0.937 | 0.997 |
| 24hpi vs 96hpi | 1.00 | 1.00 |
| 48hpi vs 72hpi | 1.02 | 0.999 |
| 48hpi vs 96hpi | 1.09 | 0.994 |
| 72hpi vs 96hpi | 1.07 | 0.998 |

**Table S10: Pairwise comparisons of viral RNA kinetics in supernatants of goat MoMs infected with IC89 strain measured by RT-qPCR, using a** **linear mixed-effects model (LMEM) and estimated marginal means (emmeans)**. Pairwise comparisons between time points were estimated using emmeans. Degrees of freedom (Df) were calculated using the Kenward–Roger approximation. P values are derived from t-tests. Statistically significant differences (P < 0.05) are highlighted in grey cells.

| **Comparison** | **Df** | **p-value** |
| --- | --- | --- |
| 24hpi vs 48hpi | 59.9 | 0.87 |
| 24hpi vs 72 hpi | 60.9 | 0.005 |
| 24hpi vs 96hpi | 61.0 | <0.0001 |
| 48hpi vs 72hpi | 58.3 | 0.0131 |
| 48hpi vs 96hpi | 59.3 | <0.0001 |
| 72hpi vs 96hpi | 58.4 | <0.0001 |
